# Research on Measurement Error Distribution and Optimization Measurement Method of Clamp-On Ultrasonic Flowmeter in Downstream Pipeline Disturbance

**DOI:** 10.3390/s25134011

**Published:** 2025-06-27

**Authors:** Zhongzhi Yang, Wei Wang, Xianjie Liu, Xin Chen, Xia Li, Xiaofeng Lu

**Affiliations:** 1 Chongqing Academy of Metrology and Quality Inspection, Chongqing 401123, China; zhongzhiyang@cqu.edu.cn (Z.Y.); 20211001001z@stu.cqu.edu.cn (W.W.); 20221001009@stu.cqu.edu.cn (X.L.); dongzhonghao@cqu.edu.cn (X.C.); 2 School of Energy and Power Engineering, Chongqing University, Chongqing 400044, China; xfluke@cqu.edu.cn

**Keywords:** clamp-on ultrasonic flowmeter, flow field, industrial disturbance, flow deviation, installation position

## Abstract

Clamp-on ultrasonic flowmeters serve as an important tool for on-site testing of gas flow meters. However, its accuracy is significantly affected by the actual flow field, thus limiting its application scenarios. To address this issue, this study focuses on typical industrial disturbance structures and obtains the evolution and distribution of non-ideal flow fields downstream of disturbances through experiments and numerical simulations, as well as their effects on velocity and flow measurement errors. The results indicate that when traditional reflection or diagonal measurements were used in the downstream of disturbances, the flow deviation was largely dependent on the installation position and angle of the clamp-on ultrasonic flowmeter. This introduced significant uncertainty and bias, rendering it impossible to correct measurement results through quantitative coefficients. Utilizing a dual-channel measurement method can enhance measurement accuracy. When two sets of sensors perpendicular to each other were used to combine the reflection measurement path, the deviation fluctuation downstream of disturbances can be effectively controlled within the range of ±2%, irrespective of the installation angle. This measurement approach significantly reduced the distance limitations on the distance of the straight pipe section during the use of clamp-on ultrasonic flowmeters.

## 1. Introduction

As industrial scale continues to expand, society faces increasing pressures of energy shortages and environmental pollution [1,2]. Efficient utilization of new energy sources to replace traditional fuels represents a favorable solution. Natural gas, as a representative of clean energy, has become increasingly common in its applications [3,4]. The consumption of such gases directly affects the economic interests of consumers and production enterprises. Therefore, the demand for gas flow measurement technology and related instrument detection technology is also increasing.

However, the current detection and calibration of gas flowmeters primarily rely on remote inspection, wherein a standard flowmeter is typically serially connected with the meter under test to compare their readings and complete the calibration process [5,6,7]. But there exist significant differences between field conditions and laboratory settings, which can impact the accuracy of the measured values of the gas flowmeter being detected. Moreover, challenges such as prolonged inspection times and constraints on normal work requirements are also present. Therefore, conducting a relevant online inspection directly at the site of gas flowmeter usage holds significant importance. However, this endeavor still faces considerable challenges due to the weakness of related technologies.

With the advancement and development of techniques, ultrasonic flowmeters have emerged with advantages such as a wide measurement range and high repeatability. Compared to the fixed and good working environment of the tubular flowmeters (which can meet the installation requirements of straight pipe sections or be in a fully developed flow field space through a pre-rectifier), the application scenarios of the clamp-on ultrasonic flowmeter are more complex and varied. However, due to its characteristics of easy assembly, portability, and non-contact measurement, it is expected to be used for online detection applications. Zhang et al. [8] conducted online calibration of large-caliber electromagnetic flowmeters used for water trade settlement using clamp-on ultrasonic flowmeters and proposed measures to improve calibration accuracy. Eisenhauer et al. [9] reported that under flow disturbance conditions, the velocity deviation of single-path clamp-on ultrasonic flowmeters used for petroleum flow measurement can exceed 20%. Zhang et al. [10] proposed a design scheme for clamp-on ultrasonic flowmeters based on the cross-correlation method and studied the relationship between flow rate indication errors and parameters such as liquid flow velocity and pipe diameter. Murakawa et al. [11] addressed the significant impedance mismatch between pipeline materials and fluids, as well as the strong signal attenuation in fluids, by proposing a new signal processing method to determine the transit time difference of clamp-on ultrasonic flowmeters. Papathanasiou et al. [12] addressed the impact of flow disturbances generated by bends in pipelines on the measurement of water flow rate by clamp-on ultrasonic flowmeters, and they proposed improving the instrument installation path configuration to compensate for flow-induced disturbances.

From the above literature, it is evident that current research on clamp-on ultrasonic flowmeters primarily focuses on liquid flow measurement and trade fields, while their application and research in the gas field are severely limited. Furthermore, the measurement accuracy of ultrasonic flowmeters is significantly affected by the actual flow field. In industrial environments, many common structures can greatly interfere with fluid flow. Typical examples include single 90° bends, plane double bends, and out-of-plane bends. When clamp-on ultrasonic flowmeters are used for measurements in these scenarios, insufficiently developed flow inside the pipe can result in significant errors, thereby greatly limiting the instrument’s applicability. Under such disturbance conditions, more accurate pipeline flow can be obtained through correction. Some scholars [8,13,14,15,16] have obtained correction factors through experimental or simulation methods; more specifically, the value of the correction factor depends on the type of flow disturbance, the distance from it, the Reynolds (Re) number, etc. Ioos et al. [17] used 2D ray-tracing methods to study the effect of flow velocity profile and turbulence on the correction factor. Zhao et al. [18] used CFD as well to calculate the correction factor for a dual-path ultrasonic flowmeter, and they discussed the optimal distance from the single-elbow disturbance. Tang [19] found that for a monaural ultrasonic flowmeter used to measure air flow, the measurement deviation decreases with the increase in Re number in a long straight circular pipeline. However, the measurement deviation increases with the increase in Re number at the downstream of a single curved pipe obstruction. Martins et al. [20] found that downstream of the contraction disturbance, when Re = 3000, the flow field required a distance of approximately (10–15)D to fully recover, while when Re = 12,000, this number increased to about 30D. Similarly, at a higher Re downstream of more complex disturbances, a longer distance is required for the flow to fully develop. Piotr et al. [21] used laser anemometry methods to study the flow field distribution of water after a 90-degree bend. They found that under two different Re number conditions of 70,000 and 100,000, the average flow measurement deviations at the 0D position downstream of the disturbance were −8.75% and −9.87%, respectively, and at the 6D position were −3.08% and −4.43%, respectively. The above research showed that the turbulence intensity and the measurement error are closely related to the initial disturbance state at the fluid inlet, and tend to be consistent with the trend of Re number changes. In addition, Enayet et al. [22] and Lim et al. [23] also found a similar trend after bending pipe disturbance. However, relevant studies mainly focus on contact-type ultrasonic flowmeters, and the target medium is primarily liquid. Due to the difference in dynamic viscosity, gas flow fields differ from liquid flow fields. Until now, the inherent errors of clamp-on ultrasonic flowmeters in gas-disturbed flow fields have not been fully researched. Nevertheless, this is crucial for online gas flow measurement and flowmeter calibration work.

This paper first conducted experimental studies on the most typical disturbance scenarios in industry, using clamp-on ultrasonic flowmeters to obtain the distribution of flow deviation downstream of disturbances. Concurrently, relevant models were established, and the experimental data were compared with numerical simulation results. Based on their consistency, the paper simulated the evolution of gas flow fields and the distribution of measurement deviations under different disturbance structures and operating conditions. Moreover, targeted operational methods were proposed to reduce errors in the instrument’s practical use. This research provides a new approach for the accurate measurement of gas flow and serves as a theoretical reference for online detection of gas flowmeters.

## 2. Methods

### 2.1. Experimental System

**Figure 1:**
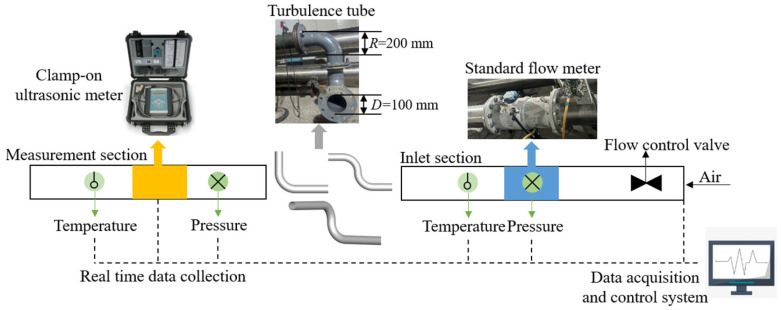
Gas flow measurement system based on the clamp-on ultrasonic flowmeter.

**Figure 2:**
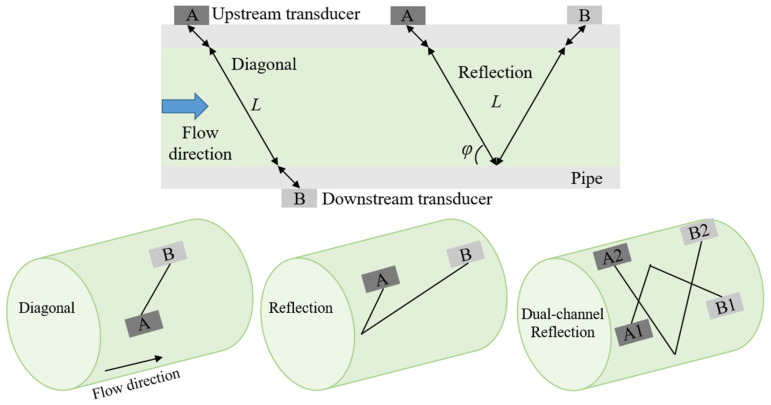
Arrangement of transducers of the clamp-on ultrasonic flowmeter.

The flow measurement test system, as shown in Figure 1, features a pipeline with a nominal diameter (D) of 100 mm, and the design of the experimental device is based on the mature air standard verification system of Chongqing Institute of Metrology and Quality Inspection. A high-precision gas turbine flowmeter (*U_r_* = 0.28%, *k* = 2) is installed at the inlet section as the standard flowmeter. The length of the inlet section is about 4 m, and the standard flowmeter is located 3 m away from the inlet. The disturbance section is installed with three different configurations of curved pipes. The double bend and out-of-plane bend are composed of two identical 90° bend pipes sealed and spliced together. The external bending radius *R* of the bend pipe is 200 mm. The measurement section is equipped with a clamp-on gas ultrasonic flowmeter (measurement uncertainty ±1–2% of reading ±0.005 m/s, repeatability 0.15% of reading ±0.005 m/s, and velocity range 0.01~25 m/s), which is used for the measurement downstream of the disturbance. The measurement data of the standard flowmeter and clamp-on ultrasonic flowmeter are collected by the control system through wired connections and camera recording, providing a basis for subsequent data analysis. Meanwhile, the standard flowmeter and clamp-on ultrasonic flowmeter are equipped with temperature and pressure measurement systems for conversion to standard flow rates, and the placement of temperature and pressure sensors complies with the requirements of the “JJG1037-2008 Turbine Flow Meter” [24] measurement technical specification. Throughout the measurement process, we conducted experiments in a laboratory equipped with a constant-temperature air conditioning system. The temperature was set at 20 °C, and the air conditioning was turned on in advance to ensure a uniform and stable ambient temperature. At the same time, the intake temperature was adjusted through a temperature-regulating device, and high-precision temperature sensors were installed in the pipeline to monitor the temperature in real time, thereby maintaining a stable medium temperature at 20 °C (±0.2 °C) during the experimental process. During the tests, the transducers of the clamp-on ultrasonic flowmeter were arranged in three different configurations: diagonal arrangement (Z-path), reflection arrangement (V-path), and dual-channel reflection arrangement (Dual-V-path), with a sound path angle of *φ* = 40°, as shown in Figure 2.

### 2.2. Numerical Simulation

**Figure 3:**
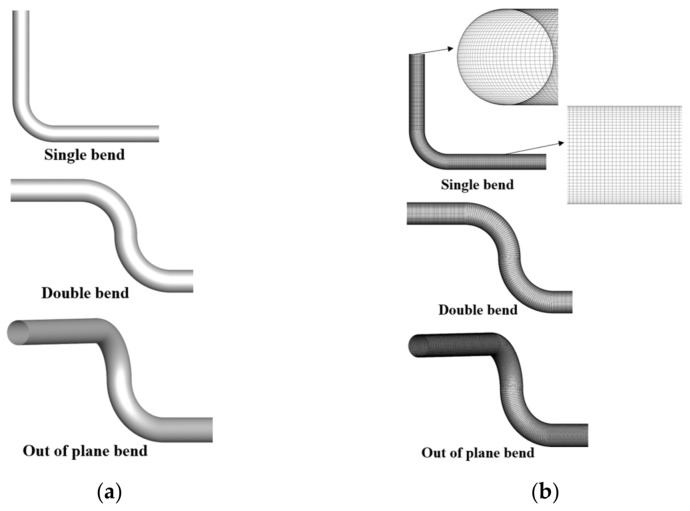
(**a**): Three types of disturbance models; (**b**): Three types of disturbance mesh.

Three types of disturbance conditions identical to the experiments were simulated in this paper; models were depicted in Figure 3a. The length of the inlet pipe segment is 8D, and a fully developed profile [25,26], calculated in a separate simulation, is specified at the inlet surface of the fluid domain, which can ensure the sufficient development of the flow profile at the upstream of the disturbance. The length of the pipeline at the downstream of the disturbance is 100D, and the inlet and outlet are set as velocity and pressure conditions, respectively. The mesh, as shown in Figure 3b, with mainly cubical elements with an edge size of D/30, is used for the discretization of the simulated fluid domain. The details of the inlet and boundary layer are also shown in Figure 3b; prism layers with a stretching factor are used for the resolution of the boundary layer near the wall, with the aim of achieving *y*^+^ = *d_w_v_t_*/*v* ≈ 1 for the highest Re. In the equation, *y*^+^ represents the dimensionless wall distance and is given as the references [27,28], *d_w_* is the distance from the wall, and *v_t_* is a reference flow velocity, also called friction velocity. The grid convergence index (GCI) [29] was used to calculate the uncertainty of numerical simulation. The out-of-plane bend is taken as an example; three identical simulations of mesh with 1,580,000 elements (coarse), 3,620,000 elements (intermediate), and 5,860,000 elements (refined) were compared. The measured flow rate of the coarse mesh was over-predicted, while the refined and intermediate meshes presented a similar result with the GCI less than 10%, and reasonable number of computational meshes 3,620,000 was finally selected for the simulation. No-slip wall is used for the wall model. The method of solving the steady Reynolds-averaged Navier–Stokes (RANS) equations was adopted in this paper, and the RNG k-ε model was selected for simulation. This model takes into account the presence of turbulent eddies and provides corresponding analytical formulas for turbulent Prandtl numbers and low Reynolds number flow viscosity, which results in good simulation accuracy [30,31,32]. The RANS equations for steady, incompressible flow are typically expressed as the following equations [33,34]:

(1) $$\frac{\partial U_{i}}{\partial x_{i}}=0$$

(2) $$U_{i}\frac{\partial U_{j}}{\partial x_{i}}=-\frac{1}{\rho }\frac{\partial }{\partial x_{i}}\left[\left(P+\frac{2}{3}k\right)\delta_{ij}+\tau_{ij}+\tau_{ij}^{t}\right]$$

where *i* and *j* are *x* and *y* for a Cartesian coordinate system, *U* is average flow velocity, *P* is the average pressure, *δ_ij_* is the Kronecker delta, *k* is the turbulent kinetic energy, *τ_ij_* is the viscous stress tensor, $\tau_{ij}^{t}$ is the Reynolds stress tensor.

The clamp-on gas ultrasonic flowmeter uses the time difference method for measurement, as illustrated in Figure 2. A and B represent a pair of ultrasonic transducers, arranged upstream and downstream of the pipeline, respectively. They emit and receive signals from each other, forming an acoustic path. The propagation times of the ultrasonic waves in the fluid in the downstream (*t_AB_*) and upstream (*t_BA_*) directions are, respectively,

(3) $$t_{AB}=\frac{L}{c+v\cos\varphi }$$

(4) $$t_{BA}=\frac{L}{c-v\cos\varphi }$$

where *L* represents the length of the acoustic path, *c* represents the speed of sound, and *φ* represents the inclination angle of the acoustic path. According to the equation, the axial flow velocity of the gas can be calculated as

(5) $$v=\frac{L\left(t_{BA}-t_{AB}\right)}{2\cos\varphi t_{AB}t_{BA}}$$

when using dual-path measurement, since the ultrasonic propagation paths pass through the centerline of the pipeline, the weighting coefficients for the two paths are the same. Therefore, the measurement value of velocity is

(6) $$v=\frac{v_{1}+v_{2}}{2}$$

therefore, the gas flow rate can be obtained as

(7) Q=kAv

where *A* is the cross-sectional area of the pipe, *k* is the flow correction factor, used to compensate for the difference between the velocity estimated along the acoustic path and the surface-averaged axial flow velocity *v_sur_*, i.e.,

(8) $$k=\frac{v_{sur}}{v}=\frac{\frac{1}{A}\int_{A}v\mathrm{dA}}{\frac{1}{\cos\varphi }\frac{1}{L}\int_{L}v\mathrm{dL}}$$

from the above formula, the deviation between the gas flow rate and the reference flow rate is obtained as

(9) $$Q_{dev}=\frac{Q-Q_{sur}}{Q_{sur}}\times 100\%$$

It is important to emphasize that in practical turbulent flow processes, the flow rate varies over time, and each instantaneous velocity curve has a corresponding *k*. In actual measurements with ultrasonic flowmeters, the turbulent curve is typically described using the Nikuradse empirical formula [35]:

(10) $$v\left(r\right)=v_{\max}{\left[1-\left(\frac{r}{R}\right)\right]}^{\frac{1}{n}}$$

where *r* is the radial distance to the centerline of the pipe, *v_max_* is the maximum fluid velocity (at the center of the pipe), *R* is the radius of the pipe, and *n* is the power exponent.

In the simulation, the transit time between the transducers is obtained by integrating the velocity within each mesh along the signal propagation path; further computation yields *v_sim_*, which is equivalent to *v*. It is worth noting that under actual disturbance conditions, the measurement of gas flow rate is affected by installation effects. That is, under non-ideal flow field conditions, *k* is a function of the upstream pipe structure of the measurement section, which is the main focus of this paper.

## 3. Results and Discussion

### 3.1. The Influence of Disturbance Type on the Flow Velocity Distribution

**Figure 4:**
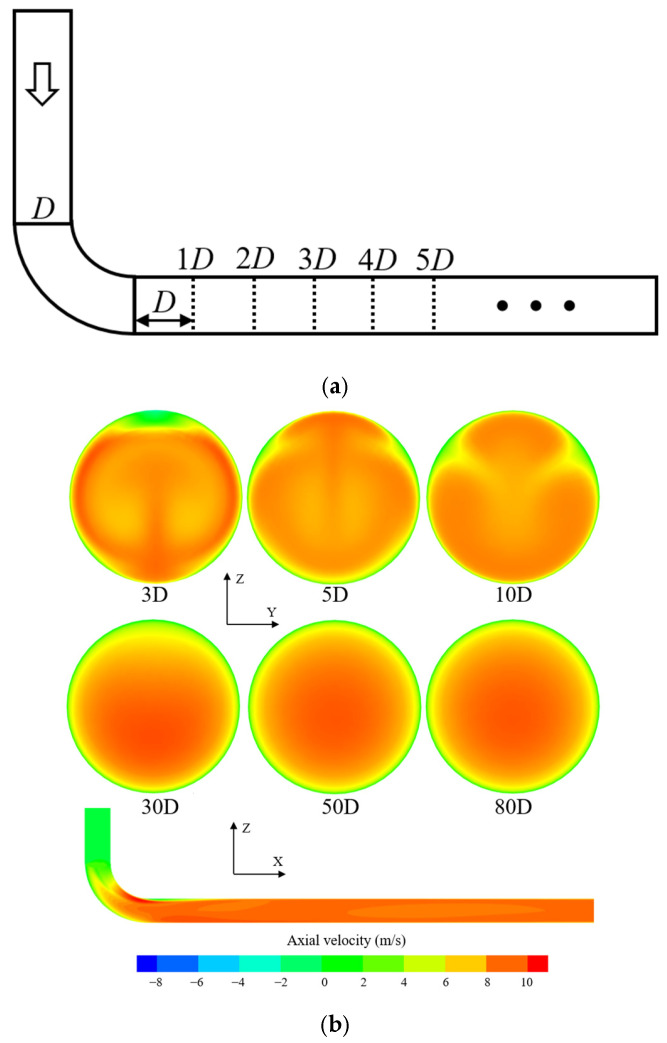
(**a**) The precise position of the bend relative to the pipeline; (**b**) variation of axial flow velocity distribution downstream of the 90° single-bend pipe with disturbance distance.

Figure 4 shows the evolution of axial flow velocity distribution downstream of the 90° single bend, as well as the precise position of the bend relative to the pipeline. The distance to the disturbance is calculated from the position where the fluid flows out of the bend, and the condition Re = 66,000 is taken as a reference. The other two types of disturbances in this section have the same downstream bend position and condition selection. It can be observed that the fluid underwent a change in flow direction after passing through the bend, resulting in a loss of kinetic energy. Inertia caused the fluid to deflect towards the outer wall direction; as a result, the flow velocity was higher near the outer wall of the bend, while it was lower near the inner wall. Within the range of (0–10)D from the disturbance, the velocity distribution was significantly distorted. It is challenging to obtain accurate data from flow meters installed within this distance. As the length of the straight pipe section increased, the degree of distortion in the velocity distribution caused by the bend gradually decreased. Beyond a distance of 10D from the disturbance, the flow field gradually returned to a stable state, and the velocity distribution within the pipe approached a central symmetric distribution typical of a straight pipe. At a distance of 30D from the disturbance, the peak axial velocity has already approached the centerline position, and at a distance of 50D, the overall velocity distribution has fully restored central symmetry.

**Figure 5:**
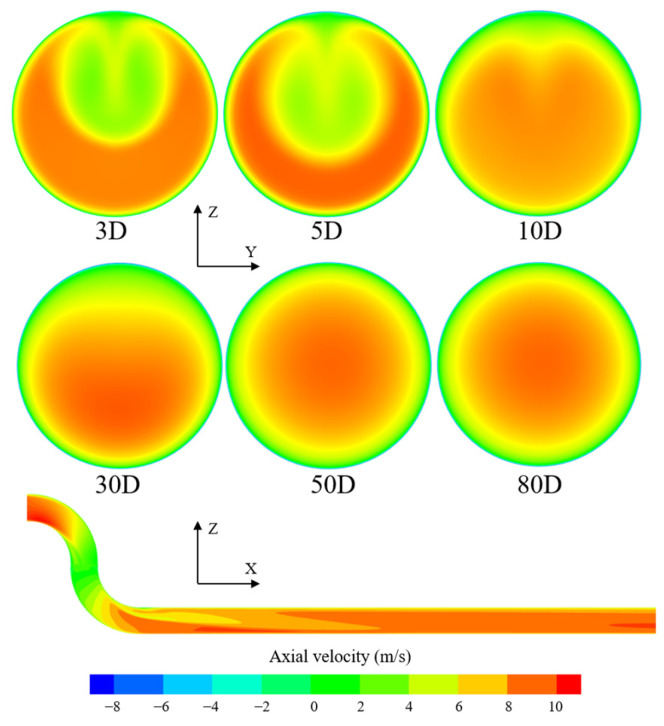
Variation of axial flow velocity distribution downstream of plane double-bend pipe with disturbance distance.

Figure 5 depicts the evolution of axial flow velocity distribution downstream of 180° plane double bends. It can be observed that due to the obstruction caused by the bent pipes, a low-speed region appeared in the upper half of the *z*-axis, resulting in an asymmetrical distribution of fluid velocity along the axis of the pipe. Compared to the velocity distribution downstream of the 90° single bend, the distortion in axial flow velocity distribution downstream of the 180° double bend was more pronounced, and the peak velocity deviation was more severe, which required a longer straight pipe section to recover. The results indicate that within the range of (0–10)D from the disturbance, the velocity distribution was significantly distorted. As the distance from the disturbance at the bend increased, the velocity in the low-speed region on the upper half of the axis gradually increased, while the velocity on both sides of the high-speed region decreased slightly. However, even at a distance of 30D from the disturbance, the velocity distribution still exhibited significant deviations from a fully developed distribution. It is only until a distance of 50D from the disturbance that the overall velocity distribution approaches central symmetry.

**Figure 6:**
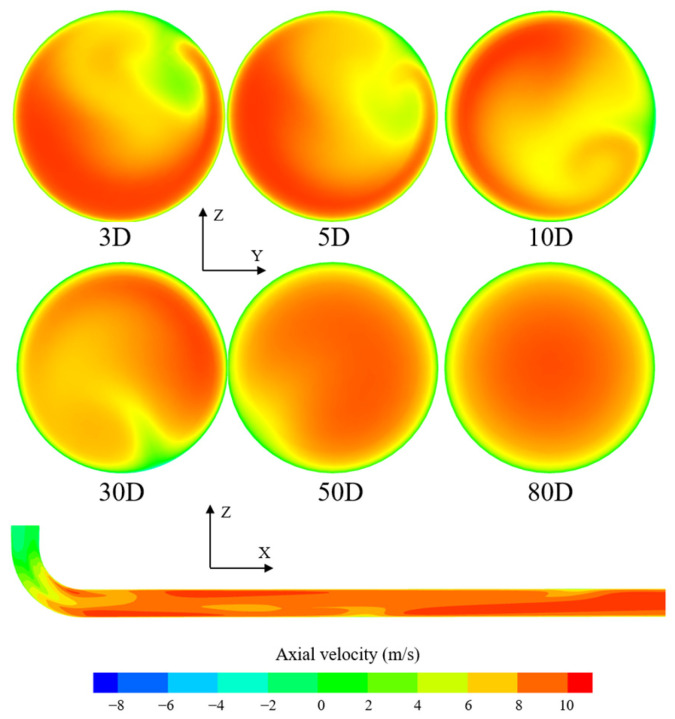
Variation of axial flow velocity distribution downstream of out-of-plane bends with disturbance distance.

Figure 6 illustrates the evolution of axial flow velocity distribution downstream of 180° out-of-plane bends. Compared to the previous two types of disturbances, after passing through a single bend in the plane, the gas streamlines remained straight, resulting in a more regular distribution pattern in the flow field. However, under the bending effect in the vertical plane, radial acceleration was generated in the gas flow field, leading to a semi-circular velocity distribution with high velocity on the outside of the pipe. Vortices are generated within the pipe system, making the distribution of sectional velocities highly asymmetrical and irregular. The results indicate that within the range of (0–30)D from the disturbance, the velocity deviation was very pronounced. Under the interaction of gas flow particles in the main flow direction, the influence of out-of-plane bending gradually diminished within the distance range of (50–80)D. It is only within a distance range approaching 80D downstream of the disturbance that the flow profile can fully recover to a fully developed state.

### 3.2. The Influence of Operating Conditions on the Flow Velocity Deviation

**Figure 7:**
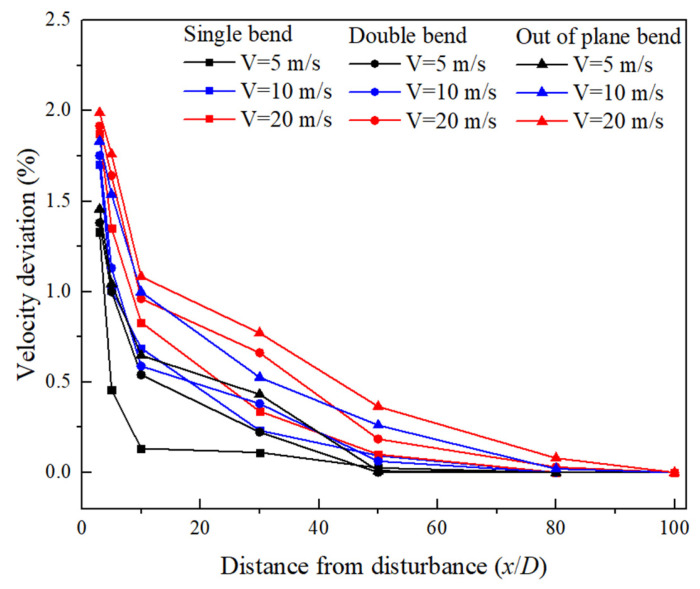
The variation curve of axial flow velocity with disturbance distance under three different flow velocities.

Figure 7 depicts the variation curve of the average deviation between the axial flow velocity along the circumferential path (the measurement path of the clamp-on ultrasonic flowmeter along the circumference of the pipeline) and the ideal turbulent flow velocity with the disturbance distance; the larger the deviation, the more significant the distortion of the flow velocity distribution will be. Results indicate that under the same type of disturbance condition, when the flow velocity was lower, the distance required for the fluid to reach a fully developed state was also shorter. The velocity gradient increased as the flow velocity increased, and the asymmetry of velocity distribution became more pronounced; the flow field required a longer distance to reach a stable state. For example, downstream of the 90° bend disturbance, when the axial velocity was 5 m/s, the distance required for the fluid to reach a fully developed state was approximately 28D, whereas when the axial velocity was 20 m/s, it required approximately 50D.

Under the same flow velocity conditions, different types of bend structures resulted in different flow disturbances. Among the three studied disturbances, out-of-plane bends exerted a greater distortion effect on gas flow, requiring a longer development space to eliminate the influence of disturbances and achieve a fully developed flow profile. For example, under the condition of the axial velocity of 20 m/s, it required a range extending beyond 80D downstream of the out-of-plane bend disturbance to reach a fully developed state. However, it is often difficult to achieve such long straight pipe lengths in industrial settings, especially under conditions of larger pipe diameters. This poses significant challenges for the measurement using clamp-on ultrasonic flow meters. Therefore, it is crucial to focus on how to achieve more accurate measurements over shorter distances.

### 3.3. The Influence of Measurement Methods on Flow Measurement Deviation

**Figure 8:**
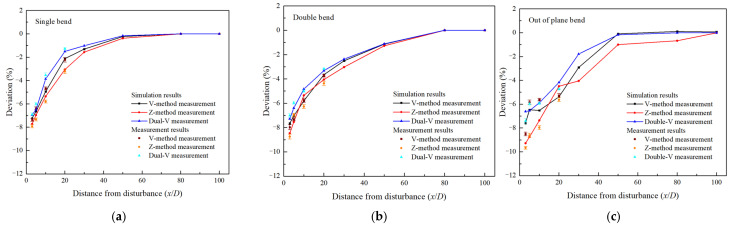
Measurement deviation distribution of three measurement methods downstream of three bend disturbances (**a**): Single bend; (**b**): Double bend; (**c**): Out-of-plane bend.

Figure 8 illustrates the experimental and simulated results of flow measurement deviations downstream of three bend disturbances (axial flow velocity V = 10 m/s, the same applies to the following), with installation paths as Z-method, V-method, and dual-channel measurement of Dual-V-method. The results indicate that within the range of (0–20)D downstream of the disturbance, for the single-bent pipe, when the Z-path of the clamp-on ultrasonic flowmeter was used, the measurement deviation varied within the range of −7.72% to −3.56%, when the V-path measurement was used, the measurement deviation ranged from −7.26% to −2.13%. For the double-bent pipe, when the Z-path of the clamp-on ultrasonic flowmeter was used, the measurement deviation varied within the range of −8.45% to −4.05%; when the V-path measurement was used, the measurement deviation ranged from −7.65% to −2.92%. The farther the distance interference, the smaller the measurement deviation of the clamp-on ultrasonic flowmeter. When the distance from the disturbance is increased to 32D and 47D, respectively, the measurement deviation under the two types of disturbances can be reduced to within 1.5%, which means that such a long straight pipe length is required to meet the measurement accuracy requirements of industry without correction. Compared to the previous two types of disturbances, the out-of-plane bend disturbance has a greater impact on the downstream measurement of the clamp-on ultrasonic flowmeter, which also leads to a more significant measurement deviation at the same distance downstream of the disturbance. When the Z-path and V-path measurements were used, the measurement deviation varied within the range of −9.62% to −5.63% and −8.16% to −5.28% in the range of (0–20)D, and it was not until the distance from interference increased to 68D that the measurement deviation could drop below 1.5%. Comparing these two commonly used measurement methods in industry, the Z-path layout has weaker resistance to vortex flow and uneven flow field distribution. In contrast, the V-path measurement not only increased the length of the sound path through reflection but also helped to avoid measurement errors caused by radial flow interference to some extent. This is because the flow velocity component Vsin*φ* along the radius direction between the two transducers can cancel each other out; as a result, compared to the Z-path method, using the V-path method resulted in higher measurement accuracy of the ultrasonic signal transit time, leading to more precise flow rate measurements. However, it should be noted that in practical applications, signal reflections off the pipe walls can cause some energy loss. Therefore, when the V-path method was used, there would be a higher requirement for the signal strength of the transducers. Moreover, results show that when the Dual-V-method was used for measurement, the measurement value represented the average of measurements in two directions, which helps to reduce errors to some extent. Under three working conditions of single bent, double bent, and out-of-plane bend, the measurement deviation ranged from −6.93% to −1.76%, −7.08% to −3.32%, and −7.38% to −4.73% in the range of (0–20)D downstream of the disturbance, which is less than those of the previous two methods. This indicates that the method can provide assistance for the measurement of clamp-on ultrasonic flowmeters downstream of disturbances.

In addition, it is worth noting that in the experiment, we noticed that the measurement performance of the clamp-on ultrasonic gas flowmeter was also affected by the characteristics of the sensor itself. During the experiment, we simultaneously used a high-frequency M-type sensor (1 MHz) and a low-frequency K-type sensor (0.5 MHz) for measurement. We found that under the same disturbance conditions, high-frequency sensors were more sensitive to small-scale eddy currents generated by bent pipes, and were also more susceptible to noise interference [36,37,38], while low-frequency sensors were less affected by changes in flow velocity profiles and could obtain more accurate measurement results downstream of disturbances. This indicates that downstream of the disturbed flow field, lower-frequency sensors should be prioritized to obtain better ultrasonic signal penetration and anti-interference ability. Meanwhile, the high-precision installation and positioning of sensors, as well as the high coupling between sensors and pipelines (relying on coupling agents), are also factors that need to be taken into account and considered in practical industrial applications. The above-mentioned content is also a component of the error between experiments and simulations. Overall, by optimizing the selection and installation arrangement of sensors during the experimental process, it can be seen that the maximum deviation between experimental and simulated results was within 1.5%, indicating that the simulation provided a good description of the flow field downstream of the disturbance.

### 3.4. The Influence of Installation Angle on Flow Measurement Deviation

The main purpose of this paper is to broaden the application scope of clamp-on gas ultrasonic flowmeters and achieve higher measurement accuracy within a short distance from the disturbance. Therefore, this section focuses on discussing the deviation variations within 10D from the disturbance. Within a relatively short range of disturbances, the vortices generated by curved pipelines, especially those as complex as out-of-plane bends, not only pose a huge challenge for measuring with clamp-on ultrasonic flowmeters, but also increase the complexity of calibrating them using simulations. In this case, the RANS model was used in this article to achieve accurate simulation of downstream disturbances to vortices and their attenuation [39], which can ensure accuracy while also considering computation time [40,41].

**Figure 9:**
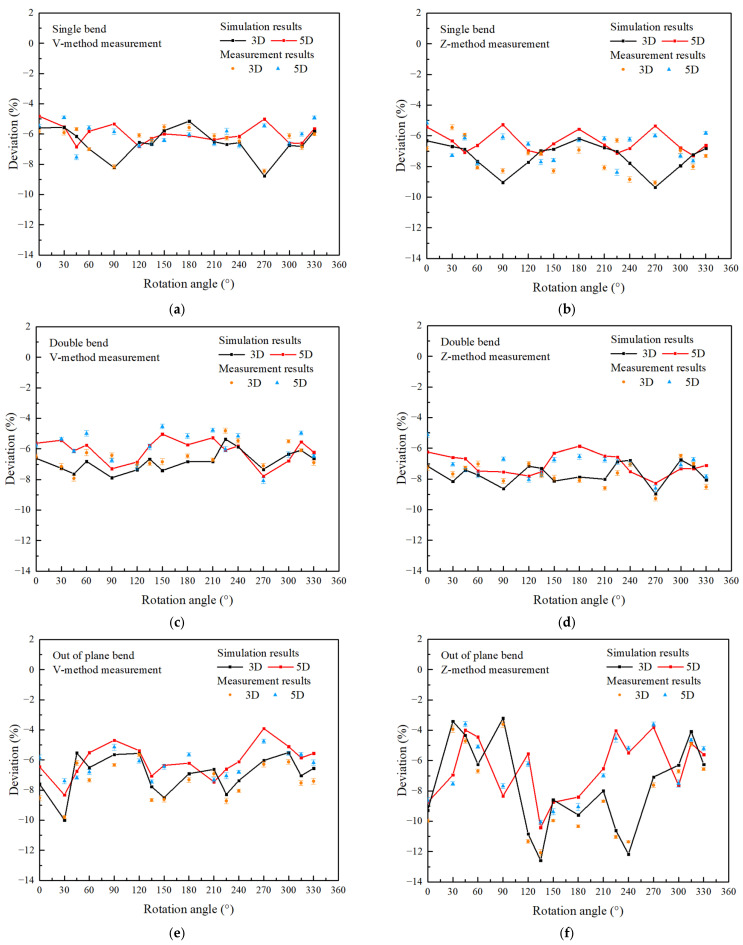
The variation of flow deviation with the rotation angle of the clamp-on ultrasonic flowmeter (**a**): Single-bend V-path; (**b**): Single-bend Z-path; (**c**): Double-bend V-path; (**d**): Double-bend Z-path; (**e**): Out-of-plane bend V-path; (**f**): Out-of-plane bend Z-path.

Figure 9 illustrates the variation of flow measurement deviation at 3D and 5D downstream of three bend disturbances with changes in the installation angle of the clamp-on ultrasonic flowmeter, and the installation paths include Z-type and V-type configurations. It can be observed that, for the single and double bents, under both Z and V installation paths, the flow deviation varied with the installation angle of the sensors. Results show that at the 3D downstream position of the single-bent disturbance, it presents a significant measurement deviation between −8.13% and −9.06% under the rotational angle of 90° and 270°. A similar phenomenon also occurred downstream of the double-bent disturbance; a significant measurement deviation between −8.27% and −9.28% under the rotational angle of 90° and 270° was observed. This indicates that when the clamp-on ultrasonic flowmeters were applied to industrial scenarios downstream of these two disturbances, it was necessary to avoid measuring positions perpendicular to the pipeline. In addition, the study also found that when avoiding these two measurement positions perpendicular to the horizontal plane, the fluctuation of measurement deviation at different rotational angles can be basically controlled within 2%. This indicates that for these two relatively simple disturbances, the downstream flow field distortion is not very complex. At a fixed distance downstream of the disturbance, as long as the measuring point perpendicular to the horizontal plane is avoided, a relatively single coefficient can be used for measurement correction, which can increase the installation flexibility of the clamp-on ultrasonic flowmeter to a certain extent.

However, unlike these two types of disturbances, the downstream flow field of out-of-plane bend disturbances was more complex and lacked clear patterns. It can be observed that, under both Z and V installation paths, the flow measurement deviations were highly dependent on the rotational angle of the installation path. At the 3D downstream position of the disturbance, the deviation fluctuated significantly within −3.63% to −12.58% and −5.52% to −9.98% under Z and V installation paths, respectively. At the 5D downstream position of the disturbance, the deviation also exhibited noticeable fluctuations within −3.59 to −10.42% and −3.93% to −7.85%. Such irregular fluctuations also indicate that the instrument cannot be quantitatively corrected for flow measurements near the disturbance in a straightforward manner. In practical measurements, both the Z-method and V-method required a different correction factor for each rotation angle. This introduced significant uncertainty and bias, posing considerable challenges to the practical application of clamp-on ultrasonic flowmeters.

**Figure 10:**
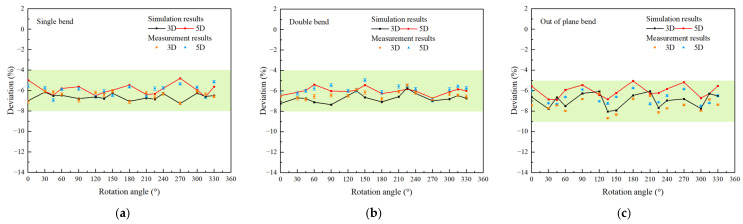
The variation of flow deviation with the rotation angle of the clamp-on ultrasonic flowmeter by dual-channel measurement (**a**): Single bend; (**b**): Double bend; (**c**): Out-of-plane bend.

Through experimentation and simulation, it is observed that the dual-channel measurement method of clamp-on ultrasonic flowmeters can improve measurement accuracy. On this basis, we conducted fitting analysis on the research results and found that with certain installation combinations, the measured values downstream of the disturbance become less sensitive to the installation rotation angle. This implies that under these conditions, high-precision measurements at relatively short distances downstream of the disturbance can be achieved through rational quantitative flow compensation correction. Figure 10 illustrates the deviation distribution when using a dual-channel measurement with two sensors arranged perpendicular to each other. It can be seen that for the two less complex disturbances of single and double bends, the fluctuation of deviation can be reduced to within 1.2% through the sensor arrangement of the Dual-V-path, and these arrangements can include measuring points perpendicular to the horizontal plane. This indicates that by combining this measurement method with a single correction factor, the measurement correction near the bend can be completed, which improves the measurement results and range to a certain extent.

What we need to emphasize here is that for out-of-plane bend with complex flow fields, the beneficial effects of this dual-channel measurement with two sensors arranged perpendicular to each other are very obvious; it can be observed that the dependence of the deviation on the rotation angle became remarkably low, with a fluctuation value of less than ±2% at the same distance from the disturbance. This indicates that with the sensor arrangement of the Dual-V-path, coupled with quantitative correction coefficients, high-precision measurements within a short distance downstream of the disturbance can be achieved. This holds significant industrial application implications for clamp-on ultrasonic flowmeters.

### 3.5. Comparison of Measurement Deviation of Different Flowmeters Downstream of Disturbance

**Figure 11:**
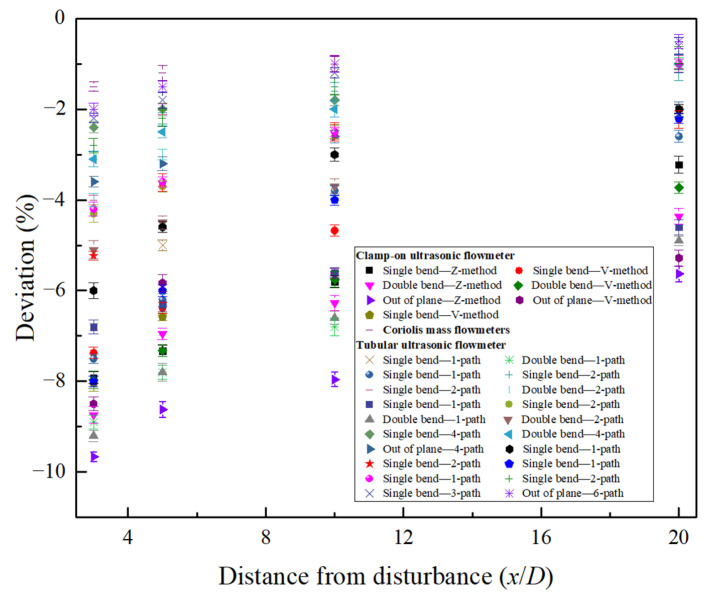
The measurement deviation distribution of the clamp-on ultrasonic flowmeter, tubular ultrasonic flowmeter, and Coriolis mass flowmeter [13,19,21,42,43,44,45,46,47,48,49,50,51].

The measurement deviation distribution of the clamp-on ultrasonic flowmeter obtained in this paper in the range of 0–20D downstream of the disturbance is compared with the measurement deviation of the ultrasonic flowmeter (1-path~6-path) [13,19,21,42,43,44,45,46,47,48] and the Coriolis mass flowmeter [49,50,51]. The results are shown in Figure 11. In the downstream of different types of disturbances, it can be found that the measurement deviation of the 1-path tubular ultrasonic flowmeter is equivalent to that of the clamp-on ultrasonic flowmeter, and the difference between the two is less than 2%. In the range of 3–5D downstream of single- and double-bend disturbance, the measurement deviation of the 2-path tubular ultrasonic flowmeter is between −2.28%~−5.15% and −3.06%~−5.23%, respectively [19,42,43,45,46,47], which is lower than that of the two measurement methods of the clamp-on ultrasonic flowmeter. Furthermore, for the 4-path and 6-path tubular ultrasonic flowmeters, the measurement deviation is less than 3% downstream of the single- and double-bend disturbance, and the measurement deviation is between −2.68% and −3.46% downstream of the out-of-plane disturbance [44,48], which is significantly lower than the measurement deviation of the clamp-on ultrasonic flowmeter. The main reason is that the multi-channel tubular ultrasonic flowmeter has a relatively complete spatial velocity capture capability. The spatial sound channels such as diamond and orthogonal paths are symmetrically arranged, and each sound channel can capture the symmetrical component of the eddy current. Through the Gaussian integral weighting calculation, it is easier to achieve the independence of the measurement for the rotation angle [13,18], and to a certain extent suppress the influence of local velocity distortion. In addition, the measurement error of the Coriolis flowmeter downstream of the disturbance can be basically controlled within the range of ±2% [49,50,51], which is also lower than the measurement error of the clamp-on ultrasonic flowmeter. The main reason is that the Coriolis flowmeter directly measures the mass flow of the fluid based on the phase difference of the vibrating tube, and measures the comprehensive effect of the whole tube section, and the dependence on the flow field is low.

Although the measurement error at a short distance downstream of the disturbance is not advantageous, the clamp-on ultrasonic flowmeter has an irreplaceable role in non-invasive installation (no need to cut pipes, no pressure loss, and no leakage risk) and industrial field comparative calibration. The research shows that it is usually not a problem to achieve high-precision measurements in the fully developed flow field (for example, more than 50D position installation) that can meet the installation requirements of the clamp-on ultrasonic flowmeter. However, because it is very sensitive to the change of the flow field, in the common industrial disturbance field, how to obtain high-precision measurement results through appropriate measurement methods and effective correction is very worthy of further discussion. In the next section, the dual-channel measurement method of two sensors arranged perpendicular to each other proposed in this paper will be experimentally verified to prove its effectiveness in complex flow fields.

### 3.6. Reference Correction Factors

Based on the results, it is found that compared with the Z-path and V-path of the clamp-on ultrasonic flowmeter commonly used in industry, the dependence of the disturbance downstream measurement deviation on the rotation angle of the sensor can be effectively reduced by two sets of mutually perpendicular sensor Dual-V-path arrangements. In this way, combined with an effective single calibration factor, the flow correction downstream of different types of disturbances can be realized; thus, the measurement specification limit of the clamp-on ultrasonic flowmeter at a close range of industrial disturbances could be broken through.

**Figure 12:**
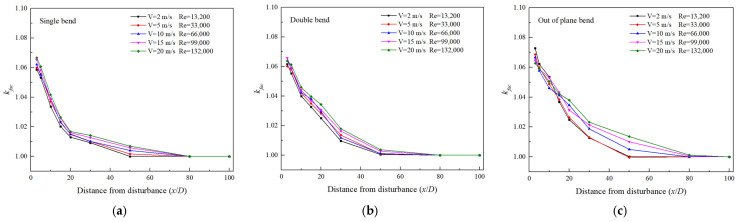
Distribution of measurement correction coefficients for Dual-V channels (**a**): Single bend; (**b**): Double bend; (**c**): Out-of-plane bend.

Figure 12 depicts the distribution of correction coefficients when using Dual-V channel measurements, where the coefficients represent the average values at different rotational angle positions. It is worth noting that within the range of Re = 13,200~132,000, the non-uniform flow field downstream of the single bend is mainly caused by the residual effects of secondary flow (vortex structures generated by the balance of centrifugal force and pressure gradient) [52,53]. The centrifugal effect increases with the increase in Re, leading to a significant increase in vortex intensity and a more chaotic downstream flow field distribution; as a result, the correction coefficient increases with the increase in Re. However, for double-bend and out-of-plane bend disturbances, the secondary flow enters the second bend without sufficient attenuation, which may cause complex vortex interactions. For example, the second bend may reconstruct or partially cancel out the vortices generated by the first bend, and the turbulent mixing is stronger at higher Re, accelerating the separation of vortex structures and reducing the dependence of residual deviation on Re [39]. Meanwhile, the turbulent energy may exceed the secondary flow energy, which dominates the reconstruction of the flow, resulting in a decrease in the contribution of the secondary flow to the distribution of the flow field. Therefore, near the disturbance exits of the double bend and out-of-plane bend, the correction coefficient fluctuates with the increase in Re, rather than monotonically increasing. Overall, the results indicate that it is easier to observe higher correction coefficient values closer to the disturbance position and higher Re conditions. In the 3D to 20D downstream range of the three disturbances, correction coefficients ranging from 1.018 to 1.072 are required to obtain more accurate flow measurement results.

In addition, based on this study, CFD 2024 and Python 2024 [54,55] can be combined to parameterize the model, medium properties, and measurement path through automatic simulation methods. The measurement deviation and correction factor can be obtained under different types of disturbances, downstream 100D range, multiple flow rate conditions, and multiple sensor rotation angles. According to the actual use requirements of the clamp-on ultrasonic flowmeter in different industrial fields, different calibration factors under the Dual-V-path arrangement of two sets of mutually perpendicular sensors are provided to compensate for the errors introduced by different interference conditions and generate the corresponding database, which can provide fast reference and matching for similar industrial scenes in the future.

**Figure 13:**
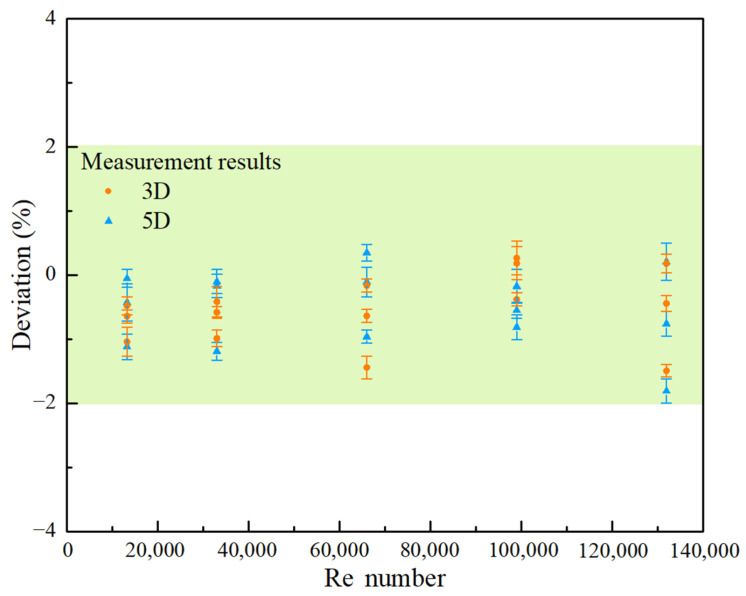
Distribution of measurement deviation under different flow conditions within the downstream range of 5D from the disturbance.

Figure 13 compares the measurement deviation distribution under different flow conditions in the 5D range downstream of the most complex out-of-plane bend disturbance in this research. During the experimental process, two sets of mutually perpendicular sensor Dual-V-path arrangements were used, and the correction coefficients obtained in Figure 12 were used for the measurement correction. It can be observed that, at different flow conditions, the experimental measurement deviations were generally within ±2%. The correction factors obtained from simulations can be effectively applied to practical measurements. This also indicates that in the practical application of clamp-on ultrasonic flowmeters, correction factors for different types of disturbances can be generated in advance through simulation calculation to compensate for non-ideal flow fields and correct flow velocities. In complex flow fields, high-precision flow measurements downstream of industrial disturbances can be achieved through a combination of multi-channel and multi-sensor methods. Relevant methods and results can save manpower and resources consumed by experiments, expanding the application scope of clamp-on ultrasonic flowmeters, which is of great significance for online gas flow measurement.

## 4. Conclusions

The clamp-on ultrasonic flowmeter is a solution for online gas flow measurement, but its measurement accuracy is significantly affected by the actual flow field. In this study, a combined approach of experiments and numerical simulations was employed to obtain the inherent error distribution of clamp-on ultrasonic flowmeters in the downstream measurement of typical industrial flow disturbances. Additionally, the study extensively discusses the influence of factors such as disturbance type, operating conditions, and measurement methods on measurement results, and proposes optimization methods for measurement accordingly. The main achievement of this work is to shorten the inlet distance when installing clamp-on ultrasonic flowmeters in typical industrial flow disturbances downstream, such as 90° single bends, double 90° plane bends, and out-of-plane bends, and expand the industrial application range of clamp-on ultrasonic flowmeters. The main conclusions are as follows:

1. In comparison to in-plane bent disturbances, out-of-plane bends exerted a greater distortion effect on gas flow. When the Z-path or V-path measurement methods were used, the flow deviation largely depended on the transducer’s rotational angle. This introduced significant uncertainty and bias, which results in the inability to adjust the measurement results through quantitative coefficients. A dual-channel approach can enhance measurement precision. When a relatively vertical Dual-V-path combination was used, the measurement values downstream of disturbances became less sensitive to the rotational angle, and the fluctuation of deviations can be effectively controlled within a range of ±2%, which enables high-precision flow rate measurements downstream of industrial disturbances.

2. In the downstream of different types of disturbances, compared with the measurement deviation of the clamp-on ultrasonic flowmeter, the measurement deviation of the 1-path tubular ultrasonic flowmeter was equivalent to that of the clamp-on ultrasonic flowmeter, and the measurement deviation of the 2-path tubular ultrasonic flowmeter was reduced. Because more channels had more complete spatial velocity capture ability, which can better suppress the influence of local velocity distortion, the measurement deviation of the downstream 4-path and 6-path tubular ultrasonic flowmeters was significantly lower than that of the clamp-on ultrasonic flowmeter. However, the dual-channel measurement method of two sensors arranged vertically with each other proposed in this paper can effectively improve the independence of the clamp-on ultrasonic flowmeter for the rotation angle in the complex flow field, so that the error can be corrected by quantitative factors, giving full play to its advantages of flexible installation and comparative calibration in the industrial field.

3. The correction factors at various distances from the disturbance were obtained through simulation and validated through experimentation, which can be used to compensate for non-ideal flow field effects. In addition, based on this study, automated simulation methods can be used to provide different calibration factors for two sets of mutually perpendicular sensor Dual-V-path arrangements according to the actual usage needs of clamp-on ultrasonic flowmeters in different industrial sites, in order to compensate for errors introduced by different interference conditions and generate corresponding databases, which can provide fast reference and matching for similar industrial scenarios in the future.

## Data Availability

Data are contained within the article.
